# The Spacing Principle for Unlearning Abnormal Neuronal Synchrony

**DOI:** 10.1371/journal.pone.0117205

**Published:** 2015-02-25

**Authors:** Oleksandr V. Popovych, Markos N. Xenakis, Peter A. Tass

**Affiliations:** 1 Institute of Neuroscience and Medicine—Neuromodulation, Jülich Research Center, Jülich, Germany; 2 Department of Neurosurgery, Stanford University, Stanford, California, United States of America; 3 Department of Neuromodulation, University of Cologne, Cologne, Germany; University of Maribor, SLOVENIA

## Abstract

Desynchronizing stimulation techniques were developed to specifically counteract abnormal neuronal synchronization relevant to several neurological and psychiatric disorders. The goal of our approach is to achieve an anti-kindling, where the affected neural networks unlearn abnormal synaptic connectivity and, hence, abnormal neuronal synchrony, by means of desynchronizing stimulation, in particular, Coordinated Reset (CR) stimulation. As known from neuroscience, psychology and education, learning effects can be enhanced by means of the spacing principle, i.e. by delivering repeated stimuli spaced by pauses as opposed to delivering a massed stimulus (in a single long stimulation session). To illustrate that the spacing principle may boost the anti-kindling effect of CR neuromodulation, in this computational study we carry this approach to extremes. To this end, we deliver spaced CR neuromodulation at particularly weak intensities which render permanently delivered CR neuromodulation ineffective. Intriguingly, spaced CR neuromodulation at these particularly weak intensities effectively induces an anti-kindling. In fact, the spacing principle enables the neuronal population to successively hop from one attractor to another one, finally approaching attractors characterized by down-regulated synaptic connectivity and synchrony. Our computational results might open up novel opportunities to effectively induce sustained desynchronization at particularly weak stimulation intensities, thereby avoiding side effects, e.g., in the case of deep brain stimulation.

## Introduction

Efficient and safe treatment requires proper dosage (see, e.g., [1–3]). Typically, an optimal dosage enables high efficacy at a minimal or at least tolerable rate of side effects. Different types of interventions require different dosage patterns. For instance, periodic regimes for non-invasive (peroral, topical etc.) drug delivery take into account washout characteristics to enable appropriate plasma levels [1–3]. Fractionation plays a major role in radiation therapy to both reduce side effects and increase therapeutic effects [4].

Deep brain stimulation (DBS) is the golden standard for the treatment of medically refractory movement disorders [5–7]. DBS protocols were empirically developed and basically advise to permanently stimulate at high frequencies (>100 Hz) at intensities close to sensory thresholds [8, 9]. The mechanism of standard high-frequency (HF) DBS is still a matter of debate [10–12]. However, HF DBS only has acute effects, i.e. neither clinical [13] nor electrophysiological [14, 15] effects persist after cessation of stimulation.

To specifically counteract abnormal neuronal synchrony by desynchronization [16], coordinated reset (CR) stimulation [17, 18] was developed. CR stimulation is a spatio-temporally patterned stimulation technique, where phase resetting stimuli are subsequently delivered to neuronal sub-populations engaged in abnormal neuronal synchronization [17, 18]. Computationally it was shown that in neuronal populations with spike timing-dependent plasticity (STDP) [19–22] CR stimulation has long-lasting effects [23–25]. In particular, CR-induced desynchronization causes a decrease of the rate of coincidences and, in turn, mediated by STDP [19–22], an unlearning of abnormal synaptic connectivity and, in turn, of abnormal neuronal synchrony [23–25]. This is achieved by the neural network being shifted from an attractor characterized by abnormal synaptic connectivity and abnormal neuronal synchrony to an attractor with physiological connectivity and synchrony [23–25].

Long-lasting CR-induced desynchronization was confirmed *in vitro* in rat hippocampal slice rendered epileptic by magnesium withdrawal [26]. In the context of Parkinson’s disease (PD), beneficial therapeutic long-lasting after-effects of electrical CR stimulation were observed in 1-methyl-4-phenyl-1,2,3,6-tetrahydropyridine (MPTP)-treated macaque monkeys, in contrast to standard HF DBS [27]. It was shown that unilateral CR stimulation delivered to the subthalamic nucleus (STN) of parkinsonian MPTP monkeys for only 2 h per day during 5 consecutive days leads to significant and sustained bilateral therapeutic after-effects for at least 30 days, while standard 130 Hz DBS had no after-effects [27]. Lasting after-effects of electrical CR stimulation of the STN were also confirmed in PD patients [28].

To develop CR neuromodulation further, we take advantage of a principle playing an important role in different research traditions including psychology, neuroscience and education. As known from pioneering studies on retrieving memories [29], experimental studies in several species addressing various aspects ranging from behavior to the underlying biochemical machinery [30–34], functional magnetic resonance imaging (fMRI) studies on face recognition [35], studies on recall of word pairs and other tasks [36–38] as well as studies in educational science [39], learning effects can be enhanced by means of the spacing principle, i.e. by delivering repeated stimuli spaced by pauses as opposed to delivering a massed stimulus (in a single long stimulation session). Another and similar principle is the pausing principle from eduction [40].

Given these findings, we hypothesize that appropriate spacing might enhance the effects of CR neuromodulation. To illustrate that the spacing principle may, in fact, boost the anti-kindling effect of CR neuromodulation, in this computational study we carry this approach to extremes. To this end, we use CR neuromodulation at particularly weak intensities which make CR ineffective if permanently delivered. We compare very weak CR neuromodulation delivered either permanently or delivered according to a spaced protocol. Intriguingly, spaced CR neuromodulation at these particularly weak intensities causes an effective anti-kindling while massed CR neuromodulation is ineffective. The spacing principle utilizes the pronounced multistability arising due to STDP. During the delivery of spaced CR neuromodulation the neuronal population successively bounces from one attractor to another one, and finally approaches an attractor characterized by down-regulated synaptic connectivity and synchrony. In this paper we first analyze the STDP-induced multistability and then continue with the specific spacing-induced effects. Finally, we discuss our findings in a broader context of DBS, non-invasive CR neuromodulation and further stimulation interventions.

## Methods

We consider a system of *N* coupled dimensionless Morris-Lecar bursting neurons [41–43]

$$\begin{array}{lll} \frac{dv_{i}}{dt} & = & -g_{ca}m_{\text{inf}}(v_{i})(v_{i}-v_{ca})-g_{k}w_{i}(v_{i}-v_{k})\\ & & -g_{l}(v_{i}-v_{l})+I_{i}^{\text{slow}}+I_{i}^{\text{const}}+I_{i}^{\text{syn}}+s_{1}I_{i}^{\text{stim}},\\ \frac{dw_{i}}{dt} & = & \phi \frac{w_{\text{inf}}(v_{i})-w_{i}}{\tau_{w}(v_{i})},\\ \frac{dI_{i}^{\text{slow}}}{dt} & = & \varepsilon_{i}\left[v^{*}-v_{i}(t-\tau )-s_{2}|I_{i}^{\text{stim}}|\right],\\ \frac{dg_{i}^{s}}{dt} & = & \frac{\alpha_{s}(1-g_{i}^{s})}{1+{\text{e}}^{-(v_{i}-\theta_{s})/\sigma_{s}}}-\beta_{s}g_{i}^{s}, \end{array}$$ (1)

with *m*_inf_(*ν*) = 0.5[1+tanh((*ν*-*ν*_1_)/ *ν*_2_)], *w*_inf_(*ν*) = 0.5[1+tanh((*ν*-*ν*_3_)/ *ν*_4_)], and *τ*_*w*_ (*ν*) = 1/cosh((*ν*-*ν*_3_)/(2*ν*_4_)). For model details and its motivation we refer to the paper [44]. $I_{i}^{\text{const}}=0.075$ is a constant current which controls the oscillatory dynamics of the neurons, $I_{i}^{\text{syn}}=N^{-1}\sum_{j}(v_{i}-v_{s})s_{ij}g_{j}^{s}(t)$ is a synaptic current received by neuron *i* due to the synaptic coupling to other neurons, and $I_{i}^{\text{stim}}$ is a stimulation current defined below. The time in Eq. (1) is given in milliseconds, and other parameters are [43, 44] *g*_*ca*_ = 1.0, *g*_*k*_ = 2.0, *g*_*l*_ = 0.5, *ν*_*ca*_ = 1.0, *ν*_*k*_ = -0.7, *ν*_*l*_ = -0.5, *ν*_1_ = -0.01, *ν*_2_ = 0.15, *ν*_3_ = 0.1, *ν*_4_ = 0.145, *ϕ* = 1.15, ν* = -0.22, *τ* = 10 ms, *α*_*s*_ = 0.1, *β*_*s*_ = 0.05, *θ*_*s*_ = 0.2, *σ*_*s*_ = 0.02, *ν*_*s*_ = -0.85, *s*_*1*_ = 0.9, and *s*_2_ = 1.0. The neurons are slightly different, where parameters *ε*_*i*_ are randomly Gaussian distributed with mean 2 × 10^-3^ and standard deviation 4 × 10^-6^ For numerical integration of model (1) we used a Runge-Kutta method of order 5(4) with adaptive step size, error tolerance 10^-4^, and maximal integration step size 0.05 ms [45].

The strength of the coupling of neuron *j* to neuron *i* is controlled by the time-dependent synaptic weight *s*_*ij*_(*t*) which is updated according to *s*_*ij*_ → *s*_*ij*_ *+ δ* Δ*s*_*ij*_, *i* ≠ *j*, with *δ* = 0.1, when the pre-synaptic neuron *j* or post-synaptic neuron *i* produces a burst. The increment Δ*s*_*ij*_ is governed by the STDP rule

$$\Delta s_{ij}=c_{\text{p}}{\text{e}}^{-|\Delta t_{ij}|/\tau_{\text{p}}}-c_{\text{d}}{\text{e}}^{-|\Delta t_{ij}|/\tau_{\text{d}}},$$ (2)

which depends on the time difference Δ*t*_*ij*_ of the nearest burst onsets of the pre- and post-synaptic neurons. The burst onsets are detected by the upward zero crossing by the membrane potential *ν*_*i*_ following an inter-burst hyperpolarization. Parameters *c*_p_ = 0.038, *c*_d_ = 0.02, *τ*_p_ = 10 ms, and *τ*_d_ = 25 ms. The synaptic weights *s*_*ij*_ are confined to the interval [*s*_min_, *s*_max_] = [0.05, 1.5] by setting *s*_*ij*_ to *s*_min_ as soon as *s*_*ij*_ tends to go below *s*_min_ via STDP or, respectively, to *s*_max_ if *s*_*ij*_ exceeds this value.

The amount of the coupling in the ensemble and the extent of synchronization among neurons are measured by the mean synaptic weight (mean coupling) $S=\frac{1}{N^{2}}\sum_{i,j}s_{ij}$ and the order parameter $R=\left|\frac{1}{N}\sum_{j}{\text{e}}^{i\psi_{j}}\right|$ [46], respectively. *ψ*_*j*_ is the phase of neuron *j* and defined as $\psi_{j}(t)=2\pi \frac{t-t_{j,n}}{t_{j,n+1}-t_{j,n}},$ *t*_*j*,*n*_ ≤ *t* < *t*_*j*,*n+1*_, where *t*_*j*,*n*_, n = 1, 2, …, are the times of the burst onsets [47].

### CR stimulation

**Fig 1:**
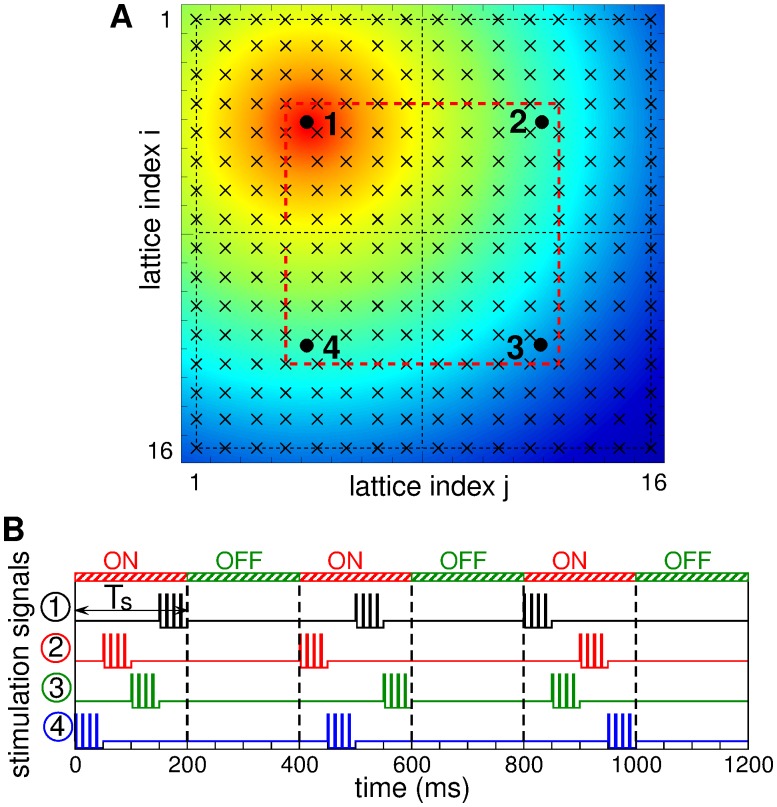
Stimulation setup of CR stimulation. (A) The neurons (marked by crosses) are arranged in a 16 × 16 2Dim lattice, and the stimulation sites (marked by black circles) are placed in the centers of the corresponding quadrants separated by black dashed lines. The spread of the current from the active site 1 for weak stimulation with intensity *c* = 0.35 is depicted by color. The square red dashed curve indicates a pathway for labeling neurons in the vicinity of stimulation sites. (B) Stimulation signals *P*_*m*_, *m* = 1,2,3,4, administered to the neuronal ensemble via each of the four stimulation sites during CR stimulation with stimulation period *T*_*s*_ = 200 ms.

The neuronal ensemble with STDP (1) is stimulated by coordinated reset (CR) stimulation [17, 18] via four stimulation sites. The *N* = 256 neurons are arranged in a 16 × 16 2Dim square lattice, and the considered four stimulation sites are placed at the center of each quadrant, as illustrated in Fig. 1A. The stimulation current administered via a stimulation site *m* decays as *c*_*n*_ exp(-2*d*_*im*_) with *c*_*n*_ = 0.588 [44] and distance *d*_*im*_ = *d*‖m-i‖ from the stimulation site *m* to the stimulated neuron *i*, where *d* = 1/16 is the lattice distance. An example of the spatial stimulation profile of the active stimulation site 1 is depicted in color in Fig. 1A. The stimulation signals consist of short high-frequency (130 Hz) trains of charge-balanced electrical pulses, as schematically illustrated in Fig. 1B. Each positive rectangular pulse of unit amplitude and length 0.2 ms is followed by a negative counterpart of length 3 ms and amplitude-1/15 with inter-pulse interval 1/130 s.

All four stimulation sites are consequently activated in a randomized way once per stimulation period of length *T*_*s*_. Activation of a stimulation site means to deliver a burst, i.e., a stimulation train of six pulses. A CR stimulus is a sequence of bursts delivered at different sites with a delay of *T*_*s*_ / 4 [Fig. 1B]. The considered stimulation period *T*_*s*_ = 200 ms approximates the oscillation period of the synchronized neurons. CR stimulation is administered intermittently, where a few periods of activated stimulation sites (ON-periods) are followed by a few periods of deactivated stimulation sites (OFF-periods), see Fig. 1B for 1:1 ON-OFF CR stimulation as in our case. In summary, the stimulation current $I_{i}^{\text{stim}}$ in Equation (1) attains the form $I_{i}^{\text{stim}}=c\sum_{m=1}^{4}c_{n}{\text{e}}^{-2d_{im}}P_{m}(t)$, where *P*_*m*_(*t*) is the above high-frequency trains of 6 charge-balanced pulses administered via stimulation site *m* when it is active and zero otherwise [Fig. 1B], and *c* is the stimulation intensity.

## Results

### STDP-induced multistability

STDP has a great impact on the intrinsic dynamics of the neuronal ensemble, in particular, by inducing a pronounced multistability. The coexisting stable states are characterized by different coupling topologies and strengths as well as by different amounts of synchronization. We illustrate the STDP-induced multistability by simulating system (1) without stimulation ($I_{i}^{\text{stim}}=0$) for different initial conditions: The individual synaptic weights *s*_*ij*_ are initially uniformly and randomly distributed in the interval [*S*_0_-*σ*, *S*_0_ + σ], and the initial values of the other variables are uniformly and randomly distributed in the interval [-0.1, 0.1]. For long enough simulation we observe that the mean synaptic weight *S*(*t*) saturates at some value and stays close to it hereafter. In Fig. 1A we plot the time courses of *S*(*t*) for 12 selected initial conditions such that 6 different regimes are realized in ensemble (1). Each of these states can attract trajectories starting from different initial conditions, which supports the stability of the established coupling regimes as revealed by the mean coupling.

**Fig 2:**
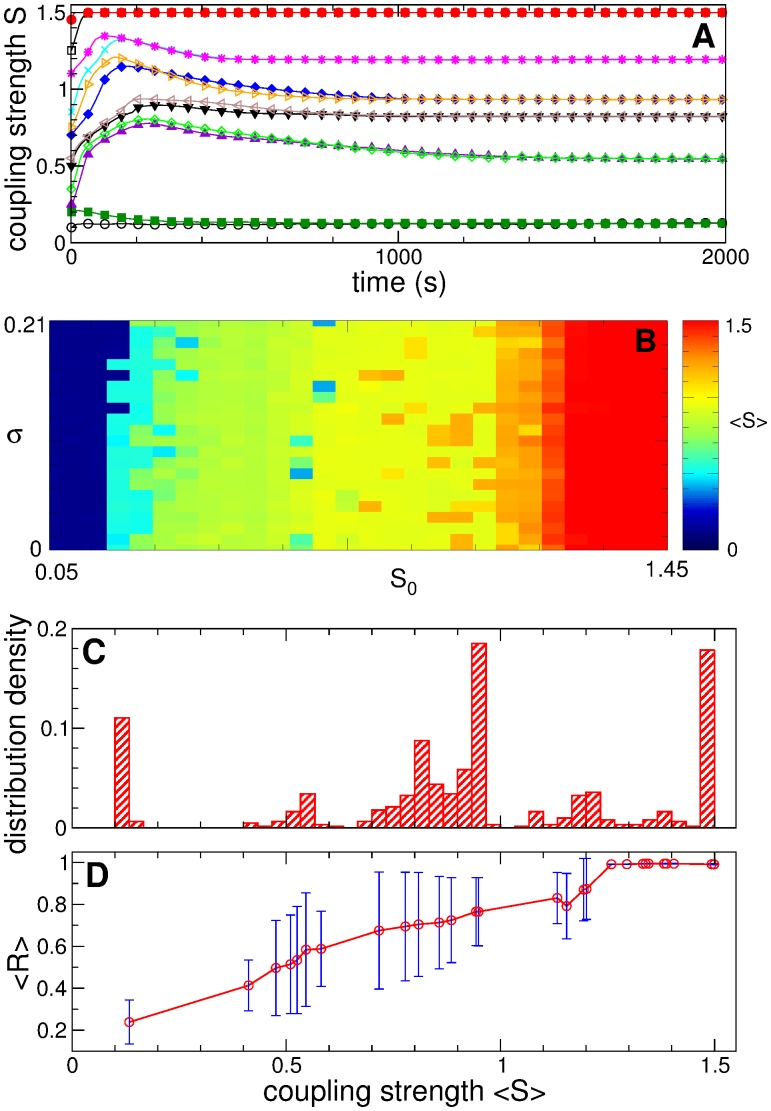
STDP-induced multistability in the neuronal ensemble (1). (A) Time courses of the mean synaptic weight *S*(*t*) for 12 different initial conditions selected from the set below. (B) Time-averaged mean synaptic weight 〈*S*〉 (encoded in color, see attached color bar) realized in the system for a set of 616 different initial conditions. For each initial condition the individual synaptic weights *s*_*ij*_(0) are uniformly and randomly distributed in the interval [*S*_0_ - σ, *S*_0_ + *σ*], where *S*_0_ = 0.05 + 0.05*k*, *k* = 1,2,…,28, and *σ* = 0.01*m*, *m* = 0,1,…,21. Other variables of ensemble (1) are uniformly and randomly distributed in the interval [-0.1, 0.1]. (C) Histogram of the distribution density of 〈*S*〉 from plot (B). (D) Time-averaged order parameter 〈*R*〉 (red circles) and standard deviation of its time fluctuations (error bars) versus the mean synaptic weight 〈S〉 of a few selected regimes established in the ensemble for the initial conditions from plot (B).

To explore possible coupling regimes that can be established in the ensemble for the mentioned initial conditions, we perform a large number of simulations by varying parameters *S*_0_ and *σ* and collect the values of the time-averaged mean synaptic weight 〈*S*〉 after skipping long enough transients. The obtained mean coupling 〈*S*〉 is depicted in color in Fig. 2B versus parameters *S*_0_ and *σ*, which illustrates a multitude of coexisting states with mean coupling ranging from small to large values. This diagram also provides an estimate of the structure of the basins of attraction of the coexisting stable states. Two main regions in Fig. 2B are the basins of attraction of the weakly coupled state [Fig. 2B, dark blue] and strongly coupled state [Fig. 2B, dark red] which are stable with respect to small variations of the initial conditions. They are complemented by the basins of attraction of other states of intermediate coupling strength. For these states, slightly different initial conditions may shift the neuronal ensemble to another regime of slightly different mean coupling, which indicates a complex structure of the phase space of the system, where the basins of attraction of the coexisting stable states are intermixed with each other. Nevertheless, an important tendency can be observed: Larger initial mean coupling *S*_0_ is expected to lead to a stronger coupling established in the ensemble due to STDP.

The volume of the basins of attraction from Fig. 2B can be quantified by calculating the frequency of occurrence of a given state. For this, we calculate the distribution density of 〈*S* 〉 obtained for the considered initial conditions and plot its histogram in Fig. 2C. The distribution clearly demonstrates three well-pronounced peaks at weak, strong, and intermediate coupling. Examples of the time dynamics of the mean coupling *S*(*t*) for such states are depicted in Fig. 2A with mean values and standard deviations, for instance, *S* = 0.123 ± 0.001 (filled green squares), *S* = 0.546 ± 0.0009 (filled violet up-triangles), *S* = 0.822 ± 0.0002 (filled black down-triangles), *S* = 0.933 ± 0.0002 (filled blue diamonds), S = 1.195 ± 0.0004 (magenta asterisks), and S = 1.499 ± 0.00003 (filled red circles). As follows from the presented values, the mean synaptic weight *S*(*t*) indeed exhibits very small time fluctuations, so that *S*(*t*) is nearly constant, and the established coupling matrices are suspected to be nearly constant as well, in particular, for large coupling.

The extent of synchronization in the neuronal ensemble depends on the amount of coupling as illustrated in Fig. 2D. The time-averaged order parameters 〈*R*〉 tends to grow as the mean coupling of the STDP-induced states increases. In other words, the neurons get more synchronized in the regime of larger mean coupling. Depending on the coupling strength the order parameter *R*(*t*) exhibits time fluctuations of different magnitude as illustrated by error bars in Fig. 2D. These fluctuations are very small for strongly coupled regimes (the order parameter is nearly constant), somewhat larger for weakly coupled regimes, and well pronounced for intermediately coupled regimes. Strongly coupled neurons are phase locked to each other, and the distribution of their phases is narrow and stationary. For the weakly coupled regimes the variation of the order parameters can be accounted for by a finite-size effect leading to the fluctuation of the order $1/\sqrt{N}$ of magnitude [48]. On the other hand, the neuronal dynamics for intermediately coupled regimes, where the order parameter is relatively large, but, nevertheless, exhibits large-amplitude oscillations, requires additional investigations. For this we consider the structure of the coupling matrices and the neuronal phase dynamics in more detail below.

**Fig 3:**
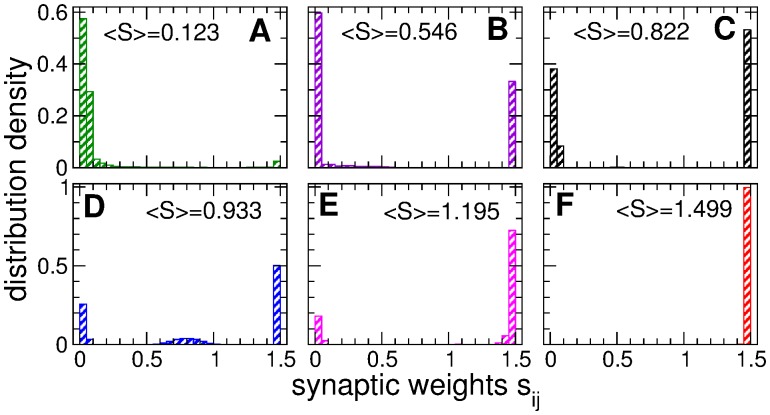
Distributions of the individual synaptic weights *s*_*ij*_ established in the neuronal ensemble (1) due to STDP. Plots (A)-(F) present the data of 6 coupling regimes illustrated in Fig. 2A. The corresponding time-averaged mean synaptic weights 〈*S*〉 are indicated in the plots.

In Fig. 3 we present distributions of the individual synaptic weights *s*_*ij*_ of the coupling regimes illustrated in Fig. 2A. The regimes of weak [Fig. 3A] and strong [Fig. 3F] coupling are characterized by uni-modal distributions of synaptic weights where the peaks of the distributions are located close to either smallest *s*_min_ or largest *s*_max_ admissible synaptic weights, respectively. If the established amount of coupling in the ensemble varies between these two limit cases, individual synaptic weights *s*_*ij*_ redistribute between these limit values, and their distributions can get bimodal [Fig. 3B,C,E] or even trimodal [Fig. 3D]. In the former cases the synapses split into two groups of very strong and very weak weights, where there practically are no synapses of intermediate strength. For the observed trimodal distribution [Fig. 3D] the emerging connections of intermediate strength are again well localized. In fact, the presented examples of the distributions of individual synaptic weights [Fig. 3] apparently demonstrate the existing underlying structure of the coupling matrices.

**Fig 4:**
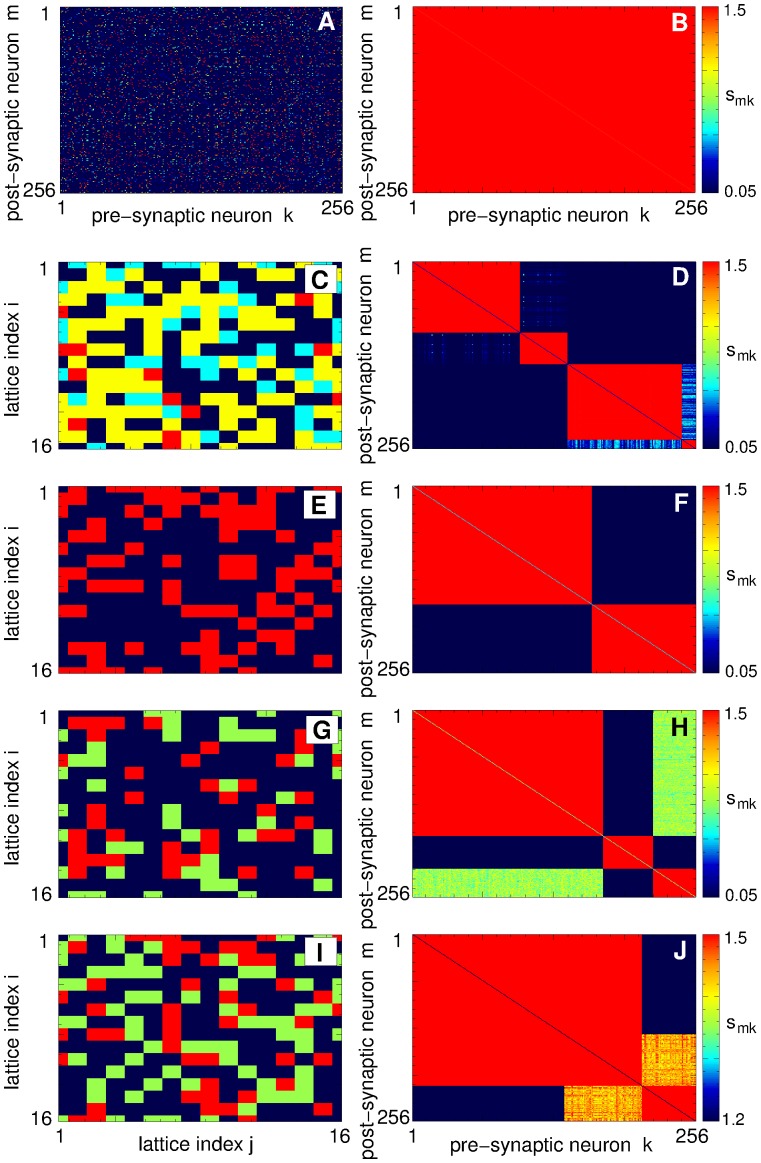
Multistability of coupling patterns established in the neuronal ensemble (1) due to STDP. In plots (A), (B), (D), (F), (H), and (J) coupling matrices are shown where the weights *s*_*mk*_ of the synapses from the pre-synaptic *k* to the post-synaptic *m* neurons are encoded in color as indicated in the attached color bars. The 16 × 16 neurons arranged in 2Dim lattice [Fig. 1A] are labeled in plots (A) and (B) as *k* = 16(*i*-1) + *j*, where *i*, *j* = 1,…,16 are the row and column indices of the lattice, respectively. Mean synaptic weight (A) 〈*S*〉 = 0.123 (see Fig. 2A, filled green squares, and Fig. 3A) and (B) 〈*S*〉 = 1.499 (see Fig. 2A, filled red circles, and Fig. 3F). The 2Dim neuronal lattices are shown in plots (C), (E), (G), and (I), where different colors depict different coupling clusters established for the mean synaptic weight 〈*S*〉 from Fig. 3B, C, D, and E, respectively. In the corresponding right plots (D), (F), (H), and (J) the corresponding coupling matrices are shown, where the neurons are labeled as above and sorted cluster-by-cluster (see text for details) according to the coupling clusters shown in the left plots.

As mentioned above, in our simulations the neurons are arranged in a square lattice of 16 × 16 elements with a total of *N* = 256 neurons [Fig. 1A]. Natural labeling of the neurons in such a 2 Dim lattice consists of two indices, *i* and *j* representing the row and the column of the lattice, respectively [Fig. 1A]. Accordingly, the coupling matrices have four indices (I, j, i′, j′) identifying a synaptic weight from the pre-synaptic neuron (*i*′, *j*′) to the post-synaptic neuron (*i*, *j*). It thus becomes difficult to illustrate such matrices in 2Dim plots. We simplify this problem by labeling the neurons in the lattice by a single index *k* according to an algorithm adopted for the sake of illustration. For example, a simple labeling can be *k* = 16(*i*-1) + *j*, *i*, *j* = 1,…,16, where the neurons are ordered “row-by-row”. Such a labeling is used in Fig. 4A, B, where the coupling matrices of weakly coupled and strongly coupled regimes are presented, respectively. The coupling matrices for these cases have a relatively simple structure, and the neurons are either weakly or strongly coupled, see also Fig. 3A, F.

The situation is more complicated for the mean coupling of an intermediate strength, where strong synapses coexist with weak or moderately strong synapses, see Fig. 3B-E. To illustrate such a cluster structure of synaptic weights we assign strongly coupled neurons to separate groups, where neurons within the same group (cluster) are coupled with the strength above a certain threshold, e.g., when *s*_*ij*_ > 1.49. In such a way several clusters of strongly coupled neurons can be detected. We depict these clusters in the 2 Dim lattice by different colors in Fig. 4C, E, G, and I. Scanning the neuronal lattice row-by-row as explained above, we label the neurons “cluster-by-cluster” such that the neurons labeled by index *k* of the first cluster are followed by the neurons of the second cluster and so on. Coupling matrices for the neurons labeled in such a way are shown in Fig. 4D, F, H, and J for the coupling regimes of intermediate strength illustrated in Fig. 3B-E, respectively.

From the shape of the coupling matrices shown in Fig. 4 it follows that neurons can form a few strongly coupled clusters with or without weak interaction between clusters [Fig. 4D, F]. The inter-cluster coupling may get somewhat stronger, but still remains much smaller than the intra-cluster coupling [Fig. 4H]. An interesting case of two-cluster coupling is illustrated in Fig. 4J, where a group of neurons from the first cluster strongly interact with the second cluster (note the range of synaptic weights in the attached color bar). In the corresponding plot in Fig. 4I of the clusters in the neuronal lattice, this particular subgroup of neurons of the first cluster is colored in green, whereas the reminder of the first cluster is shown in dark blue.

**Fig 5:**
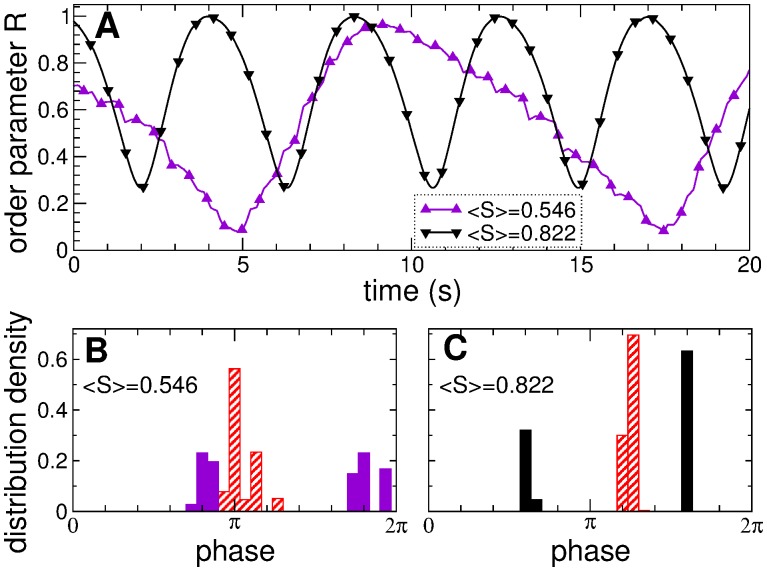
Phase dynamics of the neuronal ensemble with STDP (1) for intermediate coupling strength. (A) Time courses of the order parameter *R*(*t*) for the coupling regimes established in the ensemble due to STDP with mean synaptic weights 〈*S*〉 indicated in the legend. (B), (C) Corresponding histograms of the phase distribution density belonging to minimal (filled bars) and maximal (hatched bars) values of the order parameter from plot (A). Data shown correspond to the coupling matrices shown in Fig. 4C, D and 4E, F.

The reported cluster structure of the coupling topology strongly influences the synchronization dynamics of the neurons in the ensemble. To illustrate this effect, we calculate the phases *ψ*_*j*_ of the neurons and explore their dynamics for coupling matrices shown in Fig. 4. As mentioned above, the order parameter *R* exhibits large-amplitude oscillations for the states of intermediate coupling strength, see Fig. 2D. The time courses of *R* for the states with 〈*S*〉 = 0546 [Figs. 2A, 3B, and 4C, D] and 〈*S*〉 = 0.822 [Figs. 2A, 3C, and 4E, F] are shown in Fig. 5A. One can see that the extent of phase synchronization in the neuronal ensemble, as addressed by the values of the order parameter, strongly oscillates with relatively large periods. These oscillations originate from the time-varying distribution of the neuronal phases, where large values of the order parameter *R* reflect a narrow phase distribution, whereas a symmetric (or close to that) distribution of the phases on the unit circle leads to small values of *R*. We found that the cluster structure of the coupling matrices [Fig. 4] results in a clustering of the phases. For example, the four coupling clusters in Fig. 4D lead to two clusters of synchronized neurons: Weak inter-cluster coupling between coupling clusters 1 and 2 and between coupling clusters 3 and 4 suffices to synchronize the corresponding neurons. In such a way two groups of 140 and 116 neurons bursting at the frequencies of 5.18 Hz and 5.1 Hz, respectively, emerge. The phases of the neurons within the same synchronized cluster get narrowly distributed, and two phase clusters are formed, see Fig. 5B (filled violet histogram). Since the phase clusters run on the unit circle with different velocities corresponding to the above synchronization frequencies, they regularly meet each other and deviate apart, which causes the oscillation of the order parameter. For the considered example, the phase distributions corresponding to the minimal and maximal values of the order parameter [Fig. 5A, violet triangles] are illustrated in Fig. 5B by filled and hatched histograms, respectively. The difference between the synchronization frequencies of the neuronal clusters determines the oscillation period of the order parameter: 1/(5.18–5.1) = 12.5 s, see Fig. 5A (violet triangles).

For another example of the clustered coupling topology in Fig. 4F the situation is analogous: Two neuronal clusters of 162 and 94 synchronized neurons emerge, which burst at frequencies 4.97 Hz and 5.2 Hz, respectively. The corresponding phase clusters periodically arrange either in-phase or anti-phase with respect to each other [Fig. 5C], which causes large-amplitude oscillations of the order parameter of approximately 4.3 s period [Fig. 5A, black triangles]. Also for the coupling topology in Fig. 4G, H, where 〈*S*〉 = 0.933 [Figs. 2A and 3D], the three coupling clusters lead to two clusters of synchronized neurons (coupling clusters 1 and 3 produce a single synchronized neuronal cluster because of weak, but still sufficiently strong coupling between them, see Fig. 4H) of 211 and 45 neurons bursting at synchronization frequencies 4.82 Hz and 5.19 Hz, respectively. In general, the considered STDP rule results in a clustered structure of the coupling [Fig. 4] and synchronized neuronal dynamics, where a large coupling cluster emerges and grows in size as the mean coupling *S* increases. Finally, for large coupling strength, the neurons get all-to-all coupled [Fig. 4B] and synchronized [Fig. 2D]. In this paper we suggest an approach for shifting the neuronal ensemble from the strongly coupled and synchronized regime to the weakly coupled and desynchronized regime by a remarkably weak external simulation.

### Single-epoch CR stimulation

**Fig 6:**
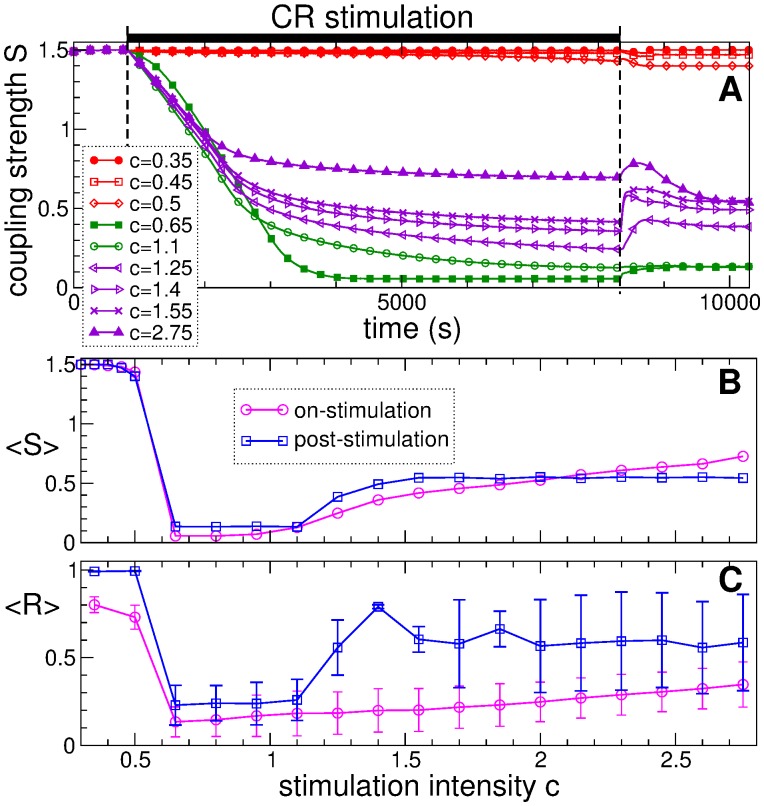
Dynamics of the mean synaptic weight and the order parameter of the neuronal ensemble with STDP (1) induced by CR stimulation. (A) Time courses of the mean synaptic weight *S*(*t*) for different CR stimulation intensities as indicated in the legend. The epoch of CR stimulation of length 7500 s is indicated by a black bar at the top of the plot. (B), (C) Time-averaged mean synaptic weight 〈*S*〉 and order parameter 〈*R*〉 established by the neuronal ensemble at the end of the CR stimulation epoch (magenta circles) and after a post-stimulation transient (blue squares) versus stimulation intensity. Error bars depict standard deviation.

The impact of CR stimulation on the neuronal ensemble with STDP is illustrated in Fig. 6. We are particularly interested in the behavior of synaptic coupling and the extent of synchronization during and after CR stimulation. It is known that CR stimulation has a desynchronizing effect on the stimulated oscillators [17, 18, 49, 50]. CR stimulation can induce an anti-kindling [23], i.e., it can initiate a process, where neuronal networks “unlearn” pathologically strong interactions and synchronization. During CR stimulation the stimulated neurons get rewired in such a way that the connectivity gets on average substantially weakened. This process is illustrated in Fig. 6A where the time course of the mean synaptic weight *S*(*t*) is shown for different stimulation intensities *c*. The population is initially strongly coupled and synchronized with 〈*S*〉 = 1.499 [compare Figs. 2, 3F, and 4B]. We show that there is a range of the CR stimulation intensity, within which a robust and long-lasting suppression of coupling and synchronization among neurons can be achieved [Fig. 6A, green curves].

We distinguish between on-stimulation and post-stimulation regimes of the ensemble during long enough CR stimulation and after long enough transients after stimulation cessation, respectively, see Fig. 6A. Accordingly, the time-averaged mean synaptic weight 〈*S*〉 and order parameter 〈*R*〉 are calculated during the last 500 s of the CR stimulation epoch and the post-stimulation transients to characterize the on- and post-stimulation regimes, respectively. The on-stimulation states just at the end of CR stimulation epoch can be considered as initial conditions for the corresponding post-stimulation states, and the on- and post-stimulation states demonstrate to a certain extent similar behavior when stimulation parameters are varied, see Fig. 6B, C. For the considered range of the stimulation intensity *c*, one can distinguish three intervals of *c* with respect to qualitatively different effects of CR stimulation on the neuronal ensemble.

- For *c* < 0.5, CR stimulation is *weak* and has no significant post-stimulation impact on the mean coupling and synchronization [Figs. 6A, red curves, and 6B, C, blue squares]. In this case, however, the synchronization is somewhat perturbed during the stimulation, but it is restored after a post-stimulation transient [Fig. 6C].
- For c ∈ (0.65,1.1), CR stimulation is *optimal* and induces maximal and sustained suppression of both coupling and synchronization [Figs. 6A, green curves, and 6B, C]. In fact, the stimulation-induced weakly coupled and desynchronized regime is preserved after the stimulation is completely switched off. For this range of the stimulation intensity, CR stimulation optimally causes a long-lasting anti-kindling.
- For *c* ≥ 1.25, the intensity of CR stimulation is *high*, which leads to a sub-optimal suppression of coupling and synchronization [Figs. 6A, violet curves, and 6B, C]. As the stimulation strength increases, the mean synaptic weight 〈*S*〉 in the on-stimulation states also increases [Fig. 6B, magenta circles], and the system converges to post-stimulation regimes of intermediate coupling strength and synchrony. The latter is relatively well suppressed during the stimulation [Fig. 6C, magenta circles], but gets stronger after cessation of stimulation [Fig. 6C, blue squares].

**Fig 7:**
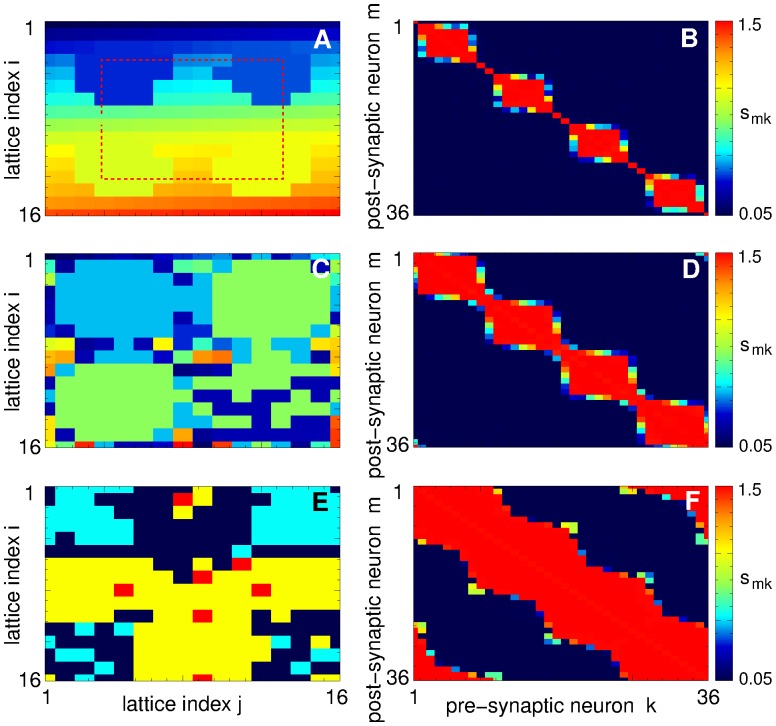
Coupling clusters induced in the neuronal ensemble with STDP (1) by CR stimulation. (A), (C), (E) 2Dim lattices of 16 × 16 neurons, where different colors depict different coupling clusters established (A) during the stimulation and (C), (E) in the post-stimulation regimes. (B), (D), (F) Individual synaptic weights *s*_*mk*_ evolving during CR stimulation among neurons labeled along the 1Dim section of the neuronal lattice, see Fig. 1A (dashed red curve) and text for details. Values of *s*_*mk*_ are encoded in color, see attached color bars. Stimulation intensity (A), (B) *c* = 0.95, (C), (D) *c* = 1.25, and (E), (F) *c* = 2.75.

To explain the observed effects of strong CR stimulation, we illustrate the coupling topology established in the neuronal ensemble during and after stimulation in Fig. 7. Already at optimal stimulation intensities, e.g., for *c* = 0.95, four coupling clusters are formed during the stimulation in the vicinity of the stimulation sites [Fig. 7A]. These clusters are visualized in Fig. 7B, where the individual synaptic weights *s*_*mk*_ from pre-synaptic to post-synaptic neurons in the vicinity of the stimulation sites are encoded in color. To obtain such a plot we considered a 1Dim section of the neuronal lattice along a square curve running through all stimulation sites, see Figs. 1A and 7A (red dashed curves). Starting at the lattice coordinate (*I*,*j*) = (8,4) the neurons are labeled along this curve running clockwise and consequently approaching stimulation sites 1, 2, 3, and 4 [Fig. 1A, red dashed curve]. Such a labeling can help to visualize and interpret the inter-neuron interactions within the same quadrant of the neuronal lattice in the vicinity of the stimulation site as well as across different quadrants assigned to different stimulation sites. The individual synaptic weights *s*_*mk*_ among 36 neurons labeled in this way are encoded in color in Fig. 7B, D, and F for on-stimulation regimes and for different stimulation strengths.

The coupling clusters induced by moderately strong, i.e., optimal CR stimulation are small and comprise neurons within the closest vicinity of the stimulation sites [Fig. 7A, B]. Moreover, these clusters do not interact in such a way that the neurons assigned to different stimulation sites do not develop connections to each other. Rather such a structure of the on-stimulation coupling topology results in a weak mean coupling [Fig. 6A, green curves], which is preserved if CR stimulation is completely switched off. The coupling matrix of the post-stimulation state after the optimal CR stimulation does not demonstrate any pronounced clustered structure whatsoever [Fig. 4A]. In such a way, the on-stimulation coupling clusters induced by optimal CR stimulation get dissolved during the post-stimulation transient, and the neuronal ensemble converges to a weakly coupled and desynchronized state.

The situation changes for strong CR stimulation. The on-stimulation coupling clusters in each quadrant grow in size and finally overlap and melt with each other [Fig. 7D, F]. Such a stimulation-induced coupling topology results in a large mean coupling of the on-stimulation states which is preserved during the post-stimulation transient [Fig. 6A, violet curves]. The post-stimulation regimes are characterized by a clustered structure of the coupling [Fig. 7C, E] which can resemble the on-stimulation coupling clusters established during CR stimulation, compare Figs. 7C and 7A. At even lager stimulation intensities the post-stimulation coupling clusters may undergo complicated transients and differ from those established during stimulation [Fig. 7E]. Nevertheless, strong CR stimulation leads to a post-stimulation clustered coupling topology and relatively large mean coupling. Interestingly, the post-stimulation coupling clusters after strong CR stimulation occupy connected domains [Fig. 7C, E] and are much less fragmented than the coupling clusters obtained for random initial conditions, see Fig. 4. As for the stimulation-free population, the post-stimulation coupling clusters lead to the emergence of clusters of synchronized neurons. For example, for the case illustrated in Fig. 7E (post-stimulation regime after CR stimulation of intensity *c* = 2.75) the four coupling clusters lead to two clusters of 146 and 110 synchronized neurons, where coupling clusters 1 and 2 [Fig. 7E, dark and light blue colors] and coupling clusters 3 and 4 [Fig. 7E, yellow and red colors] unite and result in a single synchronized neuronal cluster because of weak, but still sufficiently strong interaction between them. Such a clustering dynamics is reflected by large-amplitude oscillations of the order parameter [Fig. 6C, see also Fig. 5].

**Fig 8:**
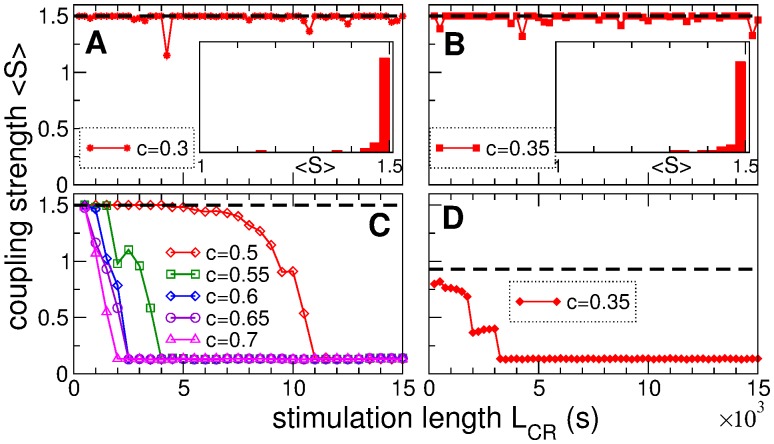
Impact of the length of CR stimulation epoch on the anti-kindling effect. The time-averaged mean synaptic weight 〈*S*〉 of the post-stimulation regimes of the neuronal ensemble with STDP (1) is plotted versus the length *L*_CR_ of a single epoch of CR stimulation. The values of the stimulation intensity *c* are indicated in the legends. CR stimulation is administered to (A)-(C) strongly coupled and (D) intermediately coupled neurons. The horizontal black dashed lines indicate the mean coupling of the initial states.

The anti-kindling effect of CR stimulation depends on the length of the stimulation epoch as illustrated in Fig. 8. The time-averaged mean synaptic weight 〈*S*〉 of the post-stimulation regimes is calculated after skipping long enough post-stimulation transients for varying length *L*_CR_ of single-epoch CR stimulation. For an optimal stimulation strength, the stimulated neuronal ensemble converges to a weakly coupled and desynchronized state if CR stimulation lasts long enough [Fig. 8C]. However, even for optimal stimulation intensity, e.g., for *c* = 0.7, CR stimulation has to be administered at least for 2000 s in order to shift the system to a weakly coupled regime. Therefore, to come up with parameters for effective CR stimulation one should find both the optimal stimulation intensity and the corresponding length of the stimulation epoch.

If CR stimulation is weak, even very long single-epoch CR stimulation does not cause any significant change of the coupling and synchronization of strongly coupled neurons, see Fig. 8A, B. In fact, the post-stimulation regime is apparently independent of the stimulation duration. Only very occasionally the observed post-stimulation regimes exhibit somewhat smaller coupling, but in most cases the neuronal ensemble returns to the initial strongly coupled state [Fig. 8A, B]. We conclude that weak CR stimulation is ineffective in inducing anti-kindling if administered to strongly coupled neurons. Note, CR stimulation of the same intensity *c* = 0.35 as in Fig. 8B can effectively decouple and desynchronize the neuronal ensemble if administered to less strongly coupled neurons, see Fig. 8D for *L*_C_ > 3000 s. The considered pre-stimulation state (the state of coupling and synchrony before the onset of CR stimulation) is characterized by 〈*S*〉 = 0.944 and has a coupling matrix similar to that shown in Fig. 4H, where three coupling clusters lead to two clusters of 213 and 43 neurons synchronized at frequencies 4.81Hz and 5.17Hz, respectively.

The presented results suggest that the notion of *weak stimulation* strongly relates to the initial conditions at stimulation onset. For example, CR stimulation of intensity *c* = 0.35 is weak and ineffective for strongly coupled neurons [Fig. 8B], whereas it is very effective in inducing an anti-kindling for intermediately coupled neurons [Fig. 8D]. It is thus reasonable to consider the notion of weak stimulation in the above broad sense, when the stimulation is ineffective in inducing the desirable effect either due to small stimulation intensity or stability properties of the stimulated system and its resistance to the external stimulation. We consider weak CR stimulation for the case when it is administered with intensity *c* = 0.35 to strongly coupled and synchronized neurons [Fig. 8B] and suggest a novel approach to improve its efficacy.

### Spaced CR stimulation

**Fig 9:**
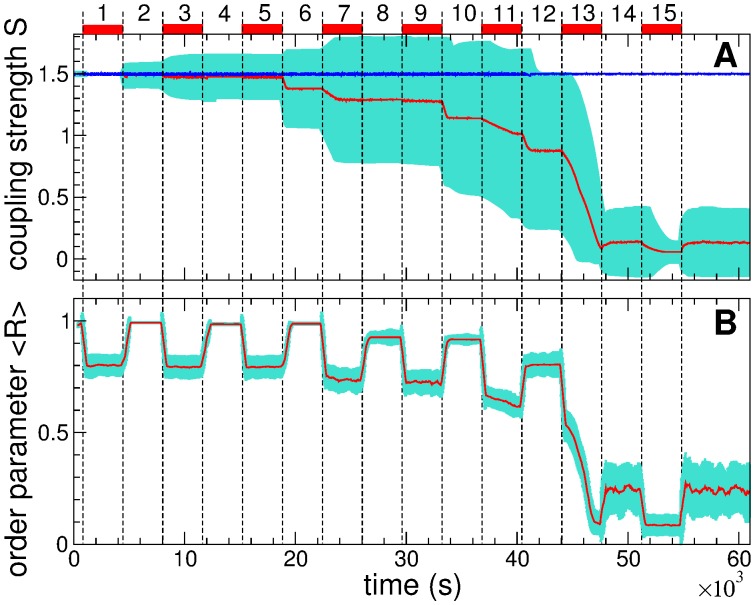
Weak spaced CR stimulation administered to the strongly coupled neuronal ensemble with STDP (1). (A) Time courses of the mean synaptic weight *S*(*t*) (red curve) and standard deviation of the individual synaptic weights *s*_*ij*_ (cyan color) across the ensemble. The mean synaptic weight for a single-epoch CR stimulation of length *L*_CR_ = 40000 s (switched off at *t* = 40800 s) is depicted by the blue curve on top. (B) Time courses of the time-averaged order parameter 〈*R*〉 (red curve) and its standard deviation (cyan color) averaged in a running window of length 500 s overlapping by 90%. Vertical dashed lines separate the enumerated stimulation- and pause-epochs, where the former are indicated by red bars on top of plot (A). Stimulation intensity *c* = 0.35, and the length of stimulation- and pause-epochs *L*_stim_ = *L*_pause_ = 3600 s.

We show that weak CR stimulation can induce anti-kindling if it is appropriately applied. Taking into account the great multistability induced in the neuronal ensemble by STDP [Fig. 2] we suggest to administer the stimulation in a spaced manner, where stimulation-epochs of CR stimulation with the stimulation signals as in Fig. 1B are recurrently followed by pause-epochs, where the stimulation is completely switched off. Time courses of the mean synaptic weight *S* and the order parameter *R* during spaced CR stimulation are illustrated in Fig. 9A and B (red curves), respectively. For long enough stimulation- and pause-epochs the neuronal ensemble converges to a weakly coupled and desynchronized regime after a few spaced stimulation cycles. The latter consist of a stimulation-epoch and the subsequent pause-epoch. In Fig. 9 the duration of the stimulation- and pause-epochs are chosen large enough, *L*_stim_ = *L*_pause_ = 3600 s, to explore and illustrate the dynamics of the coupling during each epoch. For stimulation- and pause-epochs of this duration the mean coupling saturates during many of these epochs, and the neuronal ensemble approaches the target state of weak coupling in a stepwise manner. Moreover, during each pause-epoch the system is attracted to one of the multiple coexisting stable states which may have a weaker connectivity than that of the previous pause-epoch, see pause-epochs 6, 8, 10, 12, and 14. Accordingly, as the number of stimulation-epochs increases, stimulation is delivered to a neuronal population with progressively weaker synaptic connectivity. Eventually, the spaced procedure decouples and desynchronizes neurons [Fig. 9].

**Fig 10:**
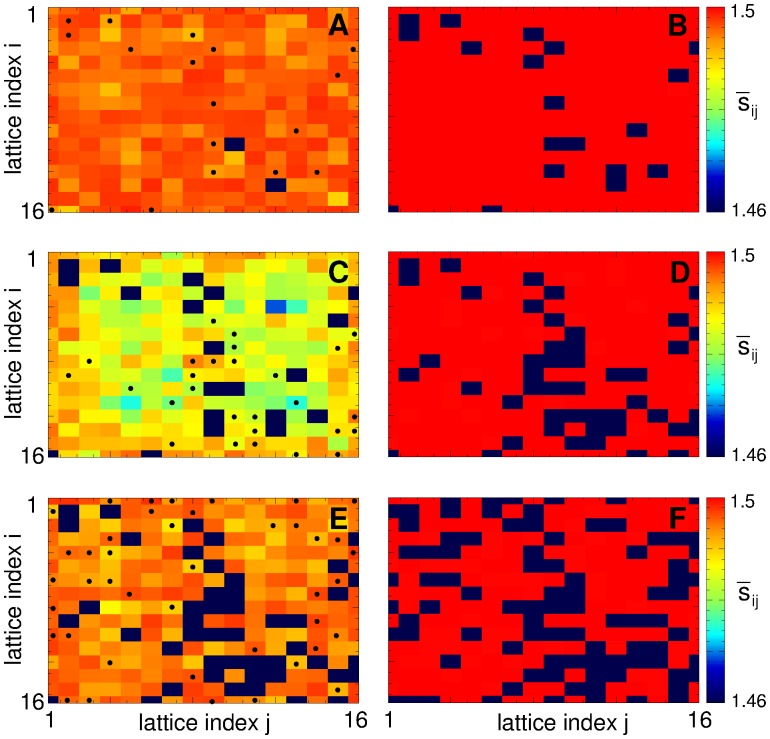
Dynamics of synaptic coupling in the neuronal ensemble with STDP (1) during weak spaced CR stimulation. The neuronal lattices are shown at the end of the stimulation-epochs (left column) (A) 5, (C) 9, and (E) 11, and pause-epochs (right column) (B) 6, (D) 10, and (F) 12 from Fig. 9. Color (except for dark blue) depicts the ensemble-averaged coupling ${\bar{s}}_{ij}$ of the corresponding neuron to the other neurons within the largest coupling cluster, whereas dark blue indicates the neurons that do not belong to this coupling cluster. Black dots in plots (A), (C), and (E) (left column) indicate neurons that decouple from the main coupling cluster during the consecutive pause-epoch (right column).

We address the mechanism of the spaced stimulation by inspecting the dynamics of the coupling in the ensemble at each transition from stimulation- to pause-epochs. The pre-stimulation state (before the first stimulation-epoch) is characterized by strong coupling such that all neurons form a single cluster with all-to-all coupling with *s*_*ij*_ = *s*_max_, see Fig. 4B. Weak CR stimulation slightly perturbs and weakens the coupling in the ensemble during stimulation-epochs. During the following pause-epochs the coupling further evolves, and some of the neurons may split (decouple) from the main (largest) coupling cluster. We illustrate such transitions in Fig. 10, where the neuronal lattices are shown at the end of a stimulation-epoch (left column) and at the end of the successive pause-epoch (right column). Color indicates the coupling that the corresponding neuron on average has to the other neurons within the largest coupling cluster. The neurons that do not belong to this coupling cluster are depicted by dark blue color.

At the end of stimulation-epoch 5, see Fig. 10A, two neurons were already separated from the main cluster (this happened at the end of pause-epoch 2, not shown), and the connections between neurons within the main cluster of 254 neurons are slightly perturbed. During the next pause-epoch 6, see Fig. 10B, 17 more neurons decouple from the main coupling cluster such that the largest coupling cluster comprises 237 neurons. At the same time the intra-cluster connectivity grows and reaches the maximal value. At the beginning of stimulation-epoch 9 the neuronal ensemble comprises a cluster of 236 strongly coupled neurons (the additional neuron with lattice coordinate (*I*,*j*) = (14,9) dropped out of the main coupling cluster during pause-epoch 8, not shown). During this stimulation-epoch the coupling among neurons within the main coupling cluster is perturbed and weakened, see Fig. 10C, but none of the neurons gets separated. Another 27 neurons decouple from the main cluster during the following pause-epoch 10, see Fig. 10D. The next decoupling transition takes place during pause-epoch 12 [Fig. 10F] resulting in a remaining coupling cluster of 166 neurons. After this transition the neuronal ensemble has intermediate coupling strength (*S* ≈ 0.876), and the next stimulation-epoch 13 of length *L*_stim_ = 3600 s shifts the system to the weakly coupled and desynchronized state [Fig. 9] in agreement with the results presented in Fig. 8D.

**Fig 11:**
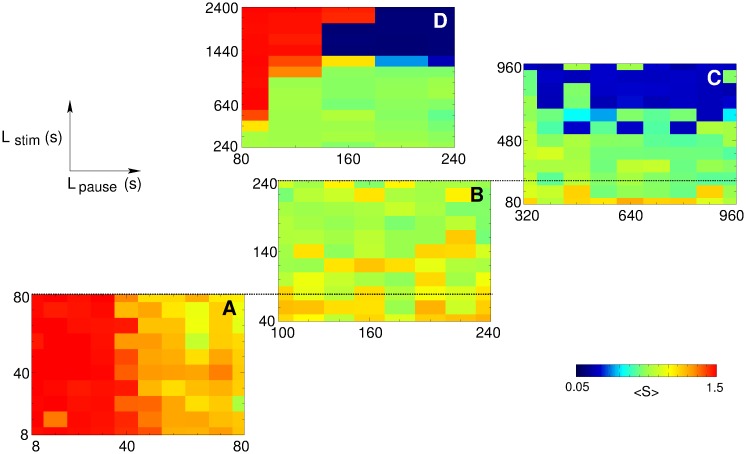
Role of stimulation- and pause-epochs in effective rewiring of the neuronal population with STDP (1) by spaced CR stimulation. Time-averaged mean synaptic weights 〈*S*〉 of the post-stimulation states are encoded in color (color bar bottom right) for four selected ranges of the lengths *L*_stim_ (vertical axes) and *L*_pause_ (horizontal axes) of the stimulation- and pause-epochs, respectively. Black dashed lines put the same values of *L*_stim_ from different plots in correspondence to each other. We did not scan the whole 2Dim (*L*_pause_, *L*_stim_)-parameter plane to reduce CPU time required and to focus on main effects.

We explore the properties of the suggested spaced protocol of CR stimulation by performing a large number of numerical simulations for varying lengths *L*_stim_ and *L*_pause_ of the stimulation- and pause-epochs, respectively. In each run the neuronal ensemble with STDP (1) starts in the strongly coupled regime and undergoes 100 cycles of length *L*_stim_ + *L*_pause_, where CR stimulation of intensity *c* = 0.35 is delivered during the stimulation-epochs only. The time-averaged mean synaptic weight 〈*S*〉 of the post-stimulation states is collected and analyzed. We found that for short pause-epochs the stimulated neurons remain strongly coupled, see Fig. 11A for *L*_pause_ < 40 s. On the other hand, if *L*_pause_ increases, the coupling in the neuronal ensemble gets weaker, see Fig. 11A for *L*_pause_ > 40 s. Hence, effective spaced CR stimulation requires pauses (i.e., pause-epochs) of sufficient length.

The coupling in the neuronal ensemble can further be suppressed for larger *L*_pause_ [Fig. 11B]. However, the stimulation-epochs also have to be of sufficient length in order to reach the states with smaller coupling [Fig. 11C]. For example, for *L*_stim_ = 80 s, spaced CR stimulation can shift the connectivities close to 0.8–0.9 only, although the length of the pause-epochs was varied up to *L*_pause_ = 960 s [Fig. 11A-C]. On the other hand, for *L*_stim_ = 560 s, the system can reach a post-stimulation state with 〈*S*〉 ≈ 0.3 already for *L*_pause_ = 480 s [Fig. 11C]. Further prolongation of the stimulation-epochs can lead the system to the desired weakly coupled and desynchronized state, for instance, with 〈S〉 ≈ 0.13 for *L*_stim_ = 1440 s and *L*_pause_ = 160 s [Fig. 11D]. However, if the stimulation-epochs are too long with respect to the pause-epochs, the spaced CR stimulation does no longer induce an anti-kindling, see Fig. 11D. Accordingly, a certain balance between the lengths of the stimulation- and pause-epochs is required for a pronounced decoupling and desynchronization.

**Fig 12:**
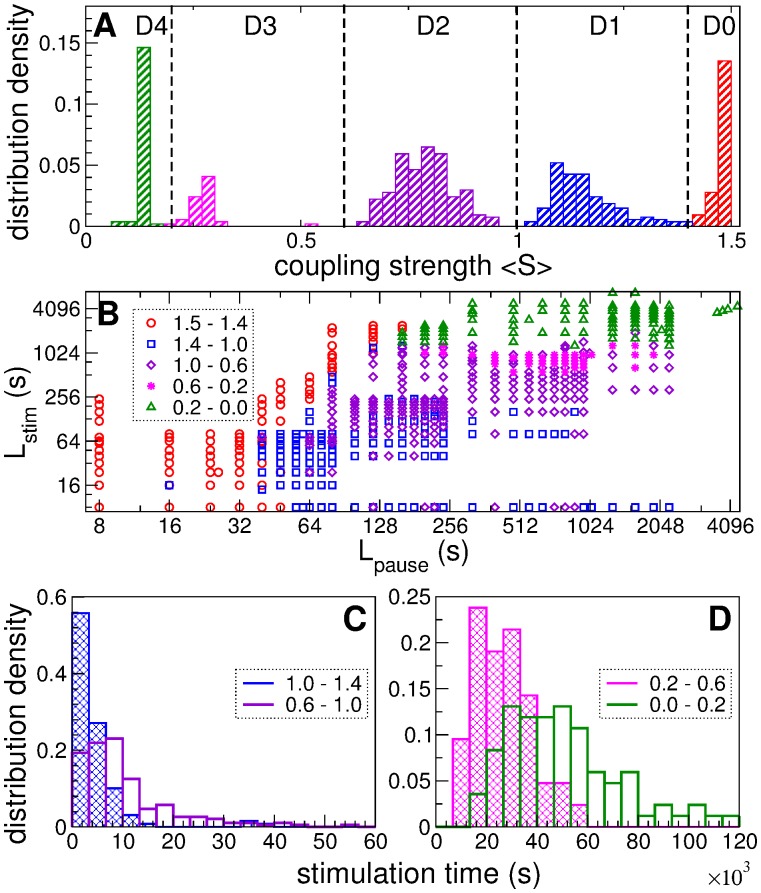
Optimizing spaced CR stimulation administered to a strongly coupled neuronal ensemble with STDP (1). (A) Histogram of the distribution density of the mean synaptic weight 〈*S*〉 collected from 540 post-stimulation states obtained by varying lengths *L*_stim_ and *L*_pause_ of stimulation- and pause-epochs, respectively. The vertical dashed lines separate 5 domains D0-D4 with respect to the magnitude of 〈*S*〉. (B) Log-log plot of parameter *L*_stim_ versus *L*_pause_ depicted by different colors depending on the domain of the corresponding post-stimulation state as indicated in the legend. (C), (D) Distributions of the stimulation time *T*_stim_ (total length of the stimulation-epochs) necessary to reach the domains (C) D1 and D2, and (D) D3 and D4 as indicated in the legends.

In Fig. 12 we summarize our findings obtained from the set of 540 simulations for different pairs (*L*_pause_,*L*_stim_) mentioned above. The distribution of 〈*S*〉 of the post-stimulation states reached by the neuronal ensemble during these simulations is shown in Fig. 12A. Based on the shape of the distribution, one can divide the entire range of the mean coupling into 5 domains: 〈*S*〉 ∈ (1.4, 1.5)—D0, 〈*S*〉 ∈ (1.0, 1.4)—D1, 〈*S*〉 ∈ (0.6, 1.0)—D2, 〈*S*〉 ∈ (0.2, 0.6)—D3, and 〈*S*〉 ∈ (0.05, 0.2)—D4. We ordered all values (*L*_p*ause*_, *L*_*stim*_) with respect to these domains and plot them in Fig. 12B. In particular, anti-kindling does not occur for insufficiently long stimulation- and pause-epochs [Fig. 12B, red circles]. Too long stimulation-epochs with respect to pause-epochs will also lead to failure of the stimulation in inducing anti-kindling, and the neuronal population stays in domain D0, i.e., it remains strongly coupled and synchronized. Even for very long pause-epochs, but short stimulation-epochs only a weak suppression of the coupling can be achieved, i.e., only transitions to D1 and, occasionally, to D2 are possible [Fig. 12B, blue squares and violet diamonds]. The tendency to reach domains D1, D2, and D3 grows as both *L*_stim_ and *L*_pause_ increase. For *L*_pause_ ≥ 160 s the transition to D4 becomes possible for appropriately large *L*_stim_. We actually found a large region of values (*L*_pause_, *L*_stim_), where weak spaced CR stimulation at intensity *c* = 0.35 decouples and desynchronizes strongly coupled neurons and reliably induces an anti-kindling [Fig. 12B, magenta asterisks and green triangles]. Note, weak single-epoch CR stimulation of such intensity can hardly shift the neuronal ensemble even to domain D1 irrespectively of the duration of stimulation, see Fig. 8A, B and 8A.

The stimulation outcome clusters in Fig. 12B, and, hence, for spaced CR stimulation we find optimal values of *L*_stim_ and *L*_pause_ for the transitions to the different domains D1–D4, respectively. An important quantity in this context is the total amount of stimulation current required. We estimate it by measuring the total stimulation time, i.e., we calculate the total length of the stimulation-epochs *T*_stim_ required before the neuronal population converged to the final state D1–D4, respectively. We thus follow the mean synaptic weight *S*(*t*), detect the time moment when it enters to and remains in a small vicinity of one of the different final states D1–D4, and calculate the total stimulation time *T*_stim_. The different final domains D1–D4 are related to different characteristic properties of the stimulation time *T*_stim_ [Fig. 12C, D]. In particular, the distribution of *T*_stim_ is skewed for D1 and D2 [Fig. 12C], while it is more symmetric for D3 and D4 [Fig. 12D]. Statistical properties of the stimulation time *T*_stim_ and the corresponding parameters *L*_pause_ and *L*_stim_ are given in Table 1.

**Table 1:** Statistics of the stimulation time at the transitions to domains D1-D4.

Domain	D1	D2	D3	D4
〈*T*_stim_〉	5141	10582	26299	52912
std *T*_stim_	11333	10623	11793	24267
median *T*_stim_	2791	7665	26026	49102
min*T*_stim_	26	131	8639	13777
at (*L*_pause_, *L*_stim_)	(8,640)	(8,880)	(960,720)	(2560,1920)
〈*L*_pause_, *L*_stim_〉	0.78	1.28	1.46	4.36

Stronger anti-kindling requires more stimulation time *T*_stim_. In fact, the mean stimulation time 〈*T*_stim_〉 necessary to reach the domains D1–D4 nearly doubles from one domain to the next with subsequently weaker coupling [Table 1]. There is a relatively broad spread of the stimulation time as given by its standard deviation and large differences between the mean and minimal values of *T*_stim_ [Fig. 12C, D]. Small *T*_stim_ provides optimal combinations of parameters *L*_stim_ and *L*_pause_, so that a given domain can be reached with a minimal amount of stimulation. Interestingly, the regimes D1 and D2 can be reached already by very short stimulation-epochs and for relatively short stimulation time *T*_stim_. Accordingly, to further optimize spaced CR stimulation one could increase the duration of the stimulation- and pause-epochs in the course of the stimulation. This might help to clear the hurdles D1–D4 at lower cost *T*_stim_. Note, effective spaced CR neuromodulation may require more total stimulation as opposed to optimal conventional (massed) CR neuromodulation. For instance, the stimulation currents administered in these two cases may relate as *c*_spaced_ *T*_stim_: *c*_opt_ *L*_CR_ = 4822: 1400, where *c*_spaced_ = 0.35 and *c*_opt_ = 0.7 are the stimulation intensities, and *T*_stim_ = 13777 s [Table 1] and *L*_CR_ = 2000 s [Fig. 8C] are the total stimulation times required to induce a pronounced anti-kindling by the considered spaced CR neuromodulation and optimal conventional CR neuromodulation, respectively.

## Discussion

In this paper we have computationally shown that the spacing principle may significantly enhance the desynchronizing effect of CR neuromodulation. In particular, we have demonstrated that the spacing principle may enable an anti-kindling [23], i.e. an unlearning of abnormal synaptic connectivity and abnormal neuronal synchrony, by using CR neuromodulation at very weak intensities which render permanently delivered CR neuromodulation ineffective. Put otherwise, even ineffective CR neuromodulation may become effective by using a spacing protocol. The CR approach fundamentally relies on the multistability of neuronal networks with STDP, which is a common phenomenon in such networks and has been observed for a variety of models and STDP rules [23–25, 51–54]. In fact, multistable dynamics is relevant to a variety of learning and memory processes in the brain and is essentially involved in the emergence of different functional and pathological regimes [55–63].

Multistability may lead to complex stimulus responses. Initially, anti-kindling was studied by using massed CR neuromodulation of sufficient intensity and duration [23]. Stimuli of this kind shift the neuronal population close to an attractor characterized by down-regulated synaptic connectivity and neuronal synchrony. Subsequent studies employed slightly modified CR protocols to probe fundamental dose-response features of CR neuromodulation:

*Transitory short-term rebound of synchrony* [51, 52]: Shorter and/or weaker CR stimuli may still be able to shift the neuronal population into the basin of attraction of a favorable attractor (with down-regulated synaptic connectivity and neuronal synchrony), but not close to the attractor. Accordingly, after cessation of stimulation the neuronal population spontaneously relaxes towards that attractor. However, on its trajectory it may undergo different types of transients, e.g., even a transitory rebound of synchrony [51, 52]. This illustrates that acute after-effects and sustained after-effects of CR neuromodulation may differ substantially (see also [64]). Accordingly, acute and sustained after-effects were separately analyzed in parkinsonian monkeys, showing that after-effects of standard high-frequency STN DBS were acute and vanished within 30 min after offset of stimulation, whereas STN CR DBS applied for only 2 h per day on 5 consecutive days led to sustained after-effects lasting for up to 30 days [27].

*Cumulative effects* [44]: In studies on CR DBS in parkinsonian monkeys [27] and PD patients [28] CR neuromodulation had to be delivered via externalized leads, since at that time no implantable pulse generator (IPG) dedicated for CR was available, so that CR stimulation had to be delivered through a portable stimulator specifically developed for this application [65]. For practical purposes stimulation via externalized leads could not be performed 24 h/day. Hence, the question arose as to whether an anti-kindling or at least long-term desynchronizing effects could be achieved by repetitively delivered CR stimuli of insufficient duration. For instance, imagine the simplified picture of a bistable system, in particular, with the dynamics being governed by a double-well potential, massed CR stimulation of sufficient duration is optimal, since during premature pauses the neuronal population relaxes back into the initial, pathological attractor state [44]. In this clinically motivated context it was computationally shown that CR neuromodulation has cumulative effects [44]: Two or more subsequently delivered short CR stimuli with appropriately bounded pauses have an effect greater than the sum of the effects of the individual CR stimuli. Cumulative effects of this kind were observed in PD patients treated with unilateral CR DBS of the STN [28]. Note, the motivation to repetitively deliver short stimuli was to come up with sort of a workaround, since for practical reasons stimuli of sufficient duration could not be delivered. In contrast, here we have shown that pauses need not degrade the desynchronizing effect of CR neuromodulation. In fact, the spacing principle may significantly enhance the efficacy of CR neuromodulation. This intriguing effect essentially relies on the STDP-induced multistability rather than bistability.

In the present study we used a network of excitatorily coupled Morris-Lecar bursting neurons with a symmetric STDP rule (Equation (2), see Ref. [44] for motivation). It would be interesting to investigate whether the spacing principle is also beneficial in networks composed of other types of model neurons and/or an asymmetric STDP rule. In our computational studies [25, 54] we have shown that CR neuromodulation can have very similar desynchronizing effects for both direct electrical stimulation and excitatory or inhibitory synaptically mediated stimulation, which was tested in neuronal ensembles with excitatory and inhibitory interactions. We therefore suspect that the presented effects of spaced stimulation could be observed for such complicated coupling and stimulation modalities, too.

Spaced CR neuromodulation might enable to further decrease the stimulation intensity of the pulses used for CR DBS and might, hence, presumably further decrease the rate of side effects caused by, e.g., current spread. Note, the total amount of stimulation current required to achieve an anti-kindling with spaced CR neuromodulation might exceed the total amount of stimulation current required for the case of optimal conventional (massed) CR neuromodulation. Put otherwise, spaced CR DBS is a strategy which might be favorable in case of, e.g., sub-optimal electrode placement or target constrains required very weak stimulation intensities, rather than a strategy to reduce the total amount of stimulation current and, hence, the battery demand.

As shown computationally, CR neuromodulation is also effective if exclusively delivered via synapses along the lines of a non-invasive approach [25, 54]. Accordingly, acoustic CR neuromodulation was developed for the treatment of chronic subjective tinnitus [54, 66]. In a proof of concept-study it was shown that acoustic CR neuromodulation causes a significant and sustained reduction of tinnitus symptoms along with a concomitant reduction of abnormal neuronal synchrony [66, 67] and abnormal effective connectivity within a tinnitus-related network of brain areas [68]. The spacing principle might also be beneficial to acoustic CR neuromodulation, for instance, by enabling to deliver this treatment at minimal loudness levels or by enabling optimized dosage patterns.

The spacing principle might also be used to improve other computationally developed desynchronizing stimulation techniques utilizing phase resetting [64, 69–72], delayed feedback [73–75] or proportional-integro-differential feedback [76] principles. However, so far, these techniques have only been applied to neuronal networks with fixed synaptic weights, and it still remains to be shown that they cause an anti-kindling in neuronal networks with STDP. In fact, uncorrelated noise delivered to a neuronal network with fixed synaptic weights is a perfect means for desynchronization, while the same stimulation pattern delivered to the same network, but equipped with STDP causes an increase of the synaptic weights and, hence, a stabilization of the neuronal synchrony [77].

In a computational study in a neuronal network without STDP it was shown that standard HF DBS may have desynchronizing effects as opposed to blocking or inhibitory effects, provided stimulation affects (nearly) exclusively excitatory targets [24]. Let us assume this holds for neuronal networks with STDP, too. In that case one might speculate whether spaced HF DBS delivered through an electrode implanted, e.g., in a fiber tract projecting onto a target population via excitatory synapses might cause an anti-kindling. In a study on closed-loop adaptive DBS (aDBS) in PD patients onsets and offsets of HF DBS were triggered by threshold crossings of the local field potential assessing beta band STN activity [78]. Effects of different stimulation protocols were studied during a few minutes of stimulus delivery. Clinical and electrophysiological (beta suppressing) effects of aDBS were significantly stronger compared to standard HF DBS as well as intermittent random DBS, where random DBS bursts were not triggered by beta LFP threshold crossings [78]. Interestingly, during aDBS on the time scale of a few minutes there was a tendency of the duration of the stimulation epochs to decrease, possibly indicative of plastic changes [78]. It might be interesting to investigate whether spaced HF DBS might be superior to intermittent random DBS (which was used as specific control for aDBS) and compare it with aDBS.

Spaced desynchronizing stimulation might, in general, serve as a control for corresponding studies or possibly even an alternative to adaptive [78] or responsive [79, 80] or demand-controlled [17, 23] brain stimulation techniques where threshold crossings are used to trigger stimulation, in particular, if the temporal specificity of closed-loop stimulation is actually not required to induce long-term plastic changes. By the same token, the spacing principle might be applied to design dosing protocols for other types of intermittent neuromodulation, e.g., vagus nerve stimulation for the treatment of epilepsies [81].

For the sake of illustration, in this study we used a simple spacing protocol with fixed duration of stimulation (*L*_stim_) and pause (*L*_pause_) epochs [Fig. 9]. However, our results [Fig. 12 and Table 1] suggest that for the neuronal network under consideration temporally non-uniform spacing protocols might be feasible and possibly even superior. In fact, we have shown that the neuronal network can be shifted from the strongly coupled and synchronized regime to intermediately coupled states [Fig. 12A, regions D1 and D2] already by means of relatively short stimulation-epochs with sufficiently long pauses, requiring only small amounts of the administered stimulation as measured by the stimulation time *T*_stim_ [Fig. 12 and Table 1]. However, a more pronounced anti-kindling [Fig. 12A, regions D3 and D4] requires much longer stimulation-epochs and pauses together with greater amounts of stimulation time *T*_stim_. Put otherwise, one might possibly use a temporally non-uniform spacing protocol where in the course of the intervention the duration of the stimulation-epochs increases. Further improvement might be obtained by means of closed-loop protocols by incorporating feedback signals appropriately reflecting the state of the network. This might eventually lead to time-dependent thresholds governing the stimulation (*L*_stim_) and pause (*L*_pause_) epochs.
